# Cerebral functional imaging using near-infrared spectroscopy during repeated performances of motor rehabilitation tasks tested on healthy subjects

**DOI:** 10.3389/fnhum.2014.00292

**Published:** 2014-05-13

**Authors:** Koji Ishikuro, Susumu Urakawa, Kouich Takamoto, Akihiro Ishikawa, Taketoshi Ono, Hisao Nishijo

**Affiliations:** ^1^System Emotional Science, Graduate School of Medicine and Pharmaceutical Sciences, University of ToyamaToyama, Japan; ^2^Department of Neurophysiotherapy, Graduate School of Medicine and Pharmaceutical Sciences, University of ToyamaToyama, Japan; ^3^Medical Systems Division, R & D Department, Shimadzu, Co. Ltd.Kyoto, Japan

**Keywords:** frontal pole, NIRS, rehabilitation, motor skill, tDCS

## Abstract

To investigate the relationship between the frontal and sensorimotor cortices and motor learning, hemodynamic responses were recorded from the frontal and sensorimotor cortices using functional near infrared spectroscopy (NIRS) while healthy subjects performed motor learning tasks used in rehabilitation medicine. Whole-head NIRS recordings indicated that response latencies in the anterior dorsomedial prefrontal cortex (aDMPFC) were shorter than in other frontal and parietal areas. Furthermore, the increment rate of the hemodynamic responses in the aDMPFC across the eight repeated trials significantly correlated with those in the other areas, as well as with the improvement rate of task performance across the 8 repeated trials. In the second experiment, to dissociate scalp- and brain-derived hemodynamic responses, hemodynamic responses were recorded from the head over the aDMPFC using a multi-distance probe arrangement. Six probes (a single source probe and 5 detectors) were linearly placed 6 mm apart from each of the neighboring probes. Using independent component analyses of hemodynamic signals from the 5 source-detector pairs, we dissociated scalp- and brain-derived components of the hemodynamic responses. Hemodynamic responses corrected for scalp-derived responses over the aDMPFC significantly increased across the 8 trials and correlated with task performance. In the third experiment, subjects were required to perform the same task with and without transcranial direct current stimulation (tDCS) of the aDMPFC before the task. The tDCS significantly improved task performance. These results indicate that the aDMPFC is crucial for improved performance in repetitive motor learning.

## Introduction

Rehabilitation ability depends on motor learning (Hanlon, 1996), and motor learning by repetitive rehabilitation task after stroke effectively improves motor functions of the upper extremity and brain neural network (Hatakenaka et al., 2007). Several studies reported activation of the prefrontal areas, supplementary motor area (SMA), premotor and motor cortices, and cerebellum when novel motor tasks were performed (Roland and Seitz, 1989; Decety et al., 1990; Friston et al., 1992; Jueptner et al., 1997; van Mier et al., 1998) or when a movement was selected based on internal or external cues (Deiber et al., 1991). However, the role of the most rostral part of the prefrontal cortex (PFC), i.e., the anterior part of the dorsomedial prefrontal cortex (aDMPFC), in motor learning remains unclear. Some imaging studies have reported that the aDMPFC is primarily activated when subjects learn new motor task(s) (Jenkins et al., 1994; Floyer-Lea and Matthews, 2004), whereas another study reported that the aDMPFC is activated by familiar rather than novel tasks (Boettiger and D'Esposito, 2005). Furthermore, previous studies have focused on acquisition of a new task, but not improving performance in the same task, which is usually achieved in motor rehabilitation. The simple test for evaluating hand function (STEF) is a standardized test for upper-extremity functions, in which the time required for completing repetitive manual tasks is measured (Yamanaka et al., 2005; Kawahira et al., 2009). In the present study, we investigated a role of the aDMPFC in performance improvement during repetitive STEF in healthy subjects using functional near infrared spectroscopy (fNIRS).

Various functional neuroimaging techniques have been developed since Roy and Sherrington (1890) reported that blood supply increases in response to neuronal activities. Studies that used positron emission tomography have reported that neuronal activities could be estimated by an increase in cerebral blood volume or flow (Fox and Raichle, 1986; Fox et al., 1988). Hemodynamic responses to neuronal activities have been measured using functional magnetic resonance imaging (fMRI) (Ogawa et al., 1993) and fNIRS (Jobsis, 1977). fNIRS can easily and non-invasively measure changes in oxy-hemoglobin (Oxy-Hb), deoxy-hemoglobin (Deoxy-Hb), and total hemoglobin (Total-Hb) based on local neuronal activities (Chance et al., 1993; Hoshi and Tamura, 1993; Kato et al., 1993; Villringer et al., 1993). Furthermore, fNIRS is sensitive to hemodynamic changes at the capillary level whereas fMRI or blood-oxygen-level dependent signals are sensitive at the small venous vessel level (Yamamoto and Kato, 2002). This suggests that compared to fMRI data, fNIRS measurements are more directly correlated to neuronal activities. In addition, fNIRS can be applied to more realistic conditions, without the body and head restrictions required in a limited space such as an fMRI environment. In fact, daily life activities, such as peeling an apple, elicited hemodynamic responses in the PFC in fNIRS measurements, but such activation was not detected for less laborious mock apple peeling that could be performed in an fMRI environment (Okamoto et al., 2004). Therefore, in the present study, we used fNIRS to measure hemodynamic activity during performance of a motor learning task used in rehabilitation medicine.

However, because fNIRS probes are usually placed on the scalp, recent fNIRS studies reported that skin blood flow in the head affected the fNIRS signals during a cognitive task (Smielewski et al., 1997; Germon et al., 1999; Takahashi et al., 2011). To dissociate cortical hemodynamic responses from skin blood flow in the forehead, we simultaneously measured the fNIRS signals with different inter-probe distances (Yamada et al., 2009) and laser Doppler tissue blood flow in the forehead. The method of using multi-distance probe arrangement enabled us to simulate a scalp-derived component of hemodynamic responses. The hemodynamic response in the aDMPFC increased in repetitive trials even when the response was corrected for its scalp-derived component. Finally, a role for the aDMPFC in performance improvement was investigated by anodal transcranial direct current stimulation (tDCS) of the aDMPFC before the trials. Anodal stimulation of tDCS has been previously applied for cortical facilitation (Brunoni et al., 2012).

## Materials and methods

### Subjects

Twelve healthy subjects (7 men, 5 women; mean age ± s.e.m., 26.4 ± 4.6 years) were used for fNIRS recordings of the whole head using a conventional head cap with 3-cm inter-probe distance (Takeuchi et al., 2009) during performance of a behavioral task using STEF (peg task; Experiment I). Because response latencies of hemodynamic responses in the aDMPFC were shorter than those in the other areas (see Results: section Hemodynamic Responses During the Peg Task), another 15 healthy subjects (7 men, 8 women; 29.1 ± 6.5 years), were enrolled for fNIRS recordings only from the aDMPFC using a head cap with different inter-probe distance during performance of the peg task as well as 2 other control tasks (Experiment II). Finally, 14 more healthy subjects (9 men, 5 women; 27.7 ± 8.2 years) participated to assess effects of tDCS of the aDMPFC on task performance (Experiment III).

All subjects were treated in strict compliance with the Declaration of Helsinki and United States Code of Federal Regulations for the protection of human participants. The experiments were conducted with the complete consent of each participant using a protocol approved by the Ethical Committee of the University of Toyama.

### Behavioral tasks

**Figure 1:**
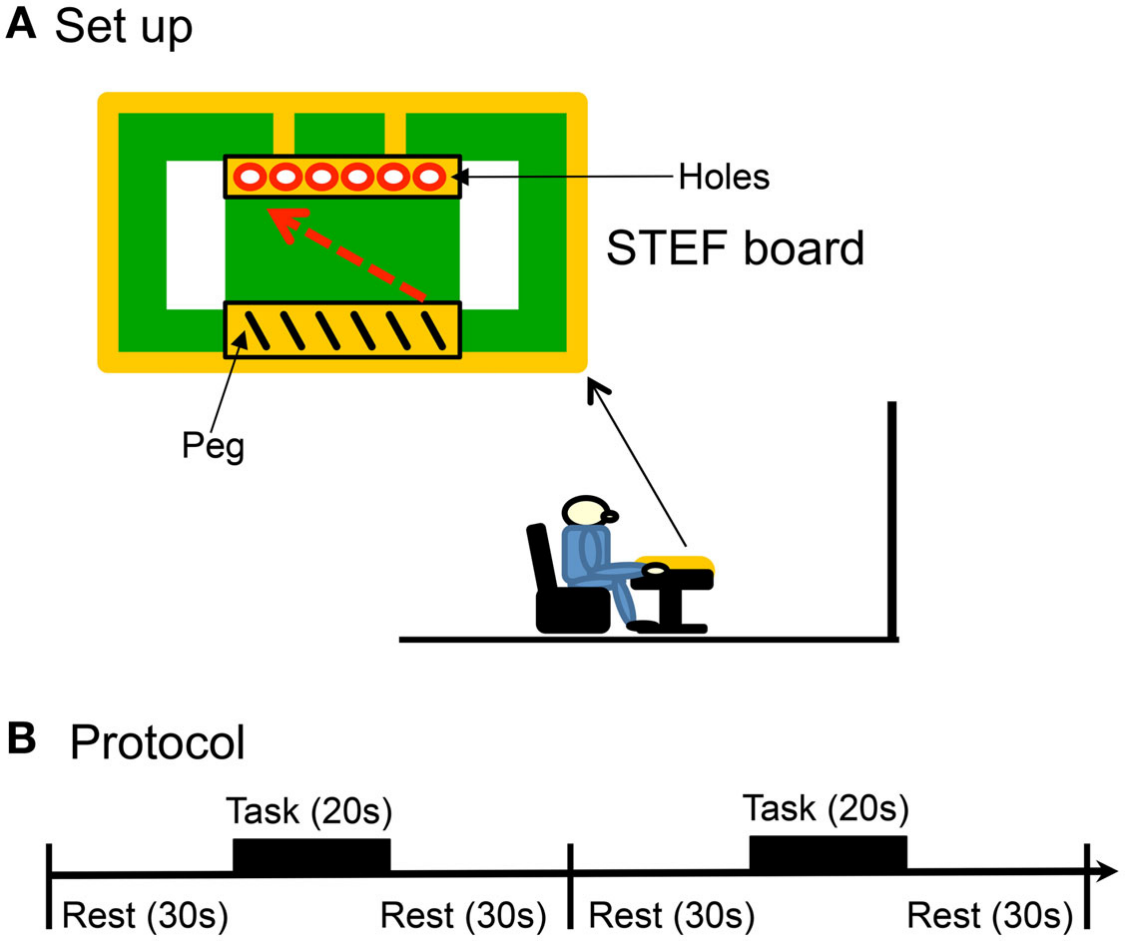
**Experimental set-up (A) and protocol (B) of the present study**. The subjects sat in a chair in front of a table, on which a board for a simple test for evaluating hand function (STEF) was placed. Each reaching and peg task consisted of 8 blocks, and each block had 3 phases: (1) rest for 30 s, (2) task for 20 s, and (3) rest for 30 s.

The subjects sat in a chair in front of a table (Figure 1A). The height of the table (RZ-800N, SAKAI med, Tokyo, Japan) was set at the height of the elbow of each sitting subject. An experimenter sat on a chair on the opposite side of the table. The subjects were instructed to perform the tasks according to the protocols of Experiments I–III (sections Experimental Protocol in Experiments I and II and Experimental Protocol of tDCS in Experiment III). Instructions for each task were given, along with a brief demonstration before each task, and the subjects were given the opportunity to practice briefly prior to the actual test.

In the peg task in which the subjects were required to perform the same behavior in the STEF, a plastic console (STEF board) was placed on the table (Figure 1A). The STEF board had a shallow rectangular dish to contain 25 metal sticks (peg, diameter, 4 mm; length, 42 mm) on one end of the board near the subject and 6-hole peg-board on the opposite end. The subjects were instructed to put their hands on the right corner of the STEF board and look at the center of the board during the rest period. During the task, they were required to pick up one of the pegs using the thumb and index finger of the right hand, put it into the hole (5 mm diameter), and not to move their trunks. The experimenter removed the peg from the hole after the subject put a peg into a hole. The subject had to put a peg into a hole sequentially from the left to right sides, and repeat the same actions from the hole in left side after putting a peg in the rightmost side. The subjects were required to repeat these actions as fast as possible for 20 s in each block of the task. Performance in the peg task was evaluated by counting the number of the pegs put into the hole (peg-score).

In the reaching task, the subjects were required to perform the same actions as the peg task without the pegs on the STEF board. In a resting task, the subjects were asked to rest without performing any task.

### Experimental protocol in Experiments I and II

The subjects were fitted the fNIRS head cap covering the whole head (Takeuchi et al., 2009) (Experiment I, *n* = 12) or a small fNIRS head cap covering the aDMPFC (Experiment II, *n* = 15; see below for more detail), and the fNIRS measurement probes (source and detector) were fixed on the head cap. Next, they were instructed on how to perform the task(s). In Experiment I, the subjects were required to perform the peg task that consisted of 8 blocks and each block consisted of 3 phases (Figure 1B): (1) rest for 30 s, (2) peg task for 20 s, and (3) rest for 30 s. In Experiment II, the subjects were required to perform 3 tasks (resting, peg, and reaching tasks), each of which consisted of 8 blocks. Each block similarly consisted of 3 phases (Figure 1B): (1) rest for 30 s, (2) resting for 20 s in the resting task, performing the peg task for 20 s, or performing the reaching task for 20 s, and (3) rest for 30 s. The start and end of the tasks were indicated by a short sound from a speaker near the STEF board. The 15 subjects in Experiment II were randomly divided into 2 groups: One group (8 subjects) sequentially performed the resting task, the peg task, and the reaching task, and the other group (7 subjects) sequentially performed the resting task, the reaching task, and the peg task. The 8 blocks in each task took place on the same day with a 5-min interval between the tasks.

### Hemodynamic response measurements using functional near-infrared spectroscopy (fNIRS) in Experiment I

**Figure 2:**
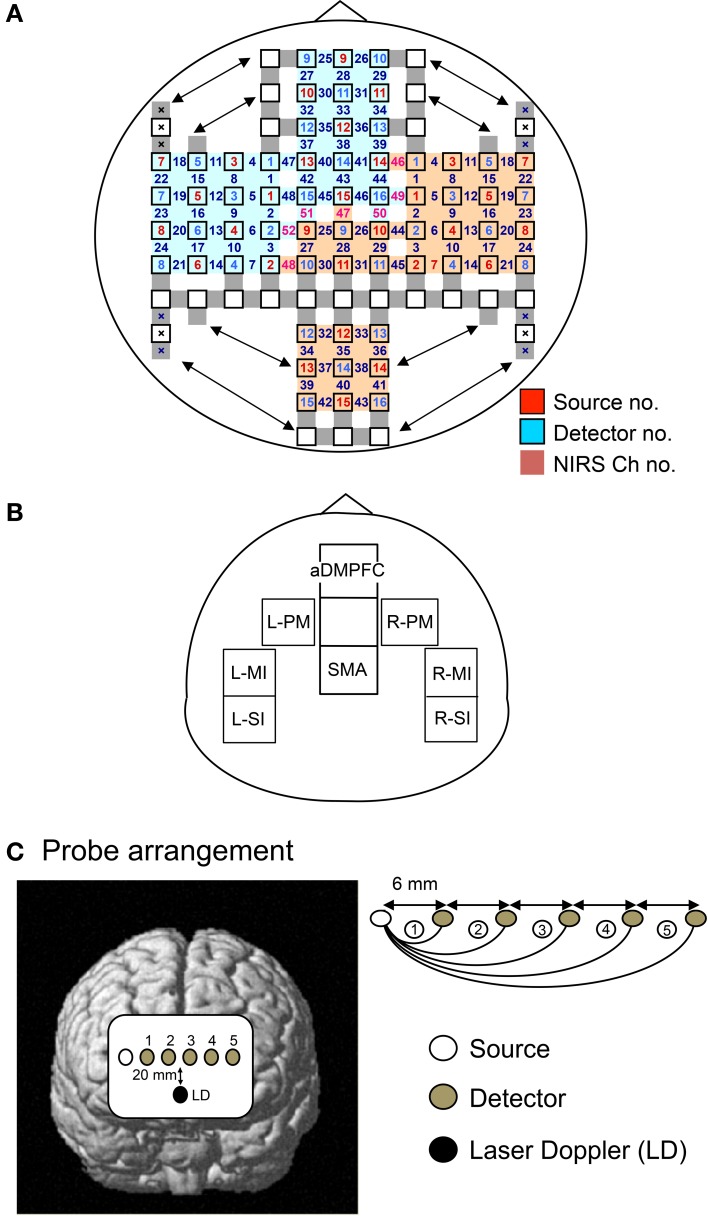
**The arrangement of probes (sources and detectors). (A,B)** Arrangement of the probes and recording channels **(A)**, and a schematic illustration of the 8 regions of interest (ROIs) **(B)** in Experiment I. aDMPFC, anterior part of the dorsomedial prefrontal cortex; SMA, supplementary motor area; L-PM, left dorsal premotor area; R-PM, right dorsal premotor area; L-MI, left hand area of the primary motor area; R-MI, right hand area of the primary motor area; L-SI, left hand area of the primary somatosensory area; R-SI, right hand area of the primary somatosensory area. **(C)** Arrangement of the probes using a linear head cap in Experiment II. The single source probe was placed on the right edge of the aDMPFC, and 5 detectors were linearly placed over the aDMPFC from the source. A laser Doppler probe was also placed 20 mm below the line of the NIRS probes.

Two fNIRS instruments (OMM 3000, Shimadzu, Co. Ltd, Kyoto, Japan) were used to examine the entire brain. The probes (source and detector) for the fNIRS measurements were fixed on a head cap (Takeuchi et al., 2009) (Figure 2A). The system consisted of 30 optical sources and 32 detectors, resulting in a total of 100 recording channels. The fNIRS sources and detector were positioned across from each other at 3-cm intervals by a new adjustment mechanism using the Guss-Bonnet theorem (Banados et al., 1994; Cummings, 2001). The depth of light penetration from the surface of the brain in adult humans has been reported to range from 0.5 to 2 cm (Fukui et al., 2003). The midpoint between the source and detector was called the “fNIRS channel.” The fNIRS source and detector detected the hemodynamic responses of these channels. Three different wavelengths (708, 805, and 830 nm) with a pulse width of 5 ms were used to detect the hemodynamic responses. The mean total irradiation power was less than 1 mW. Changes in the Hb concentration [ΔOxy-Hb, ΔDeoxy-Hb, and ΔTotal-Hb (ΔOxy Hb + ΔDeoxy Hb)] from baseline were estimated based on a modified Lambert-Beer law (Seiyama et al., 1988; Wray et al., 1988). Because continuous wave systems cannot measure optical path length (Hoshi, 2003) and no specific value for optical path length has been defined in any previous publication (e.g., Duncan et al., 1996), we adopted the unit obtained by multiplying the molar-concentration by the unknown path length (mM × cm) for our measurements. In this unit (mM × cm), the path length (cm) is supposed to be constant across time in the same channel although the path length is unknown. This indicates that the resultant changes in this unit (mM × cm) across time reflect changes in concentration of Oxy-Hb, Deoxy-Hb, or Total-Hb (mM).

After recording, the 3-D location of the fNIRS probes was measured using a Digitizer (Real NeuroTechnology Co. Ltd., Japan) with reference to the nasion and bilateral external auditory meatus. The location of each fNIRS channel was determined by converting the actual coordinates to Motoreal Neurological Institute (MNI) coordinates (Ye et al., 2009). In some subjects, the locations of NIRS channels were confirmed by stereotaxic superimposition on the surface of the reconstructed 3-D MRI brain image obtained for that subject.

### Hemodynamic response measurements in Experiment II

The alternative head cap (linear head cap) was designed to differentiate between brain and scalp hemodynamic responses (Figure 2C). Six probes (a single source and 5 detectors) were linearly placed over the aDMPFC; the single source probe was placed at the right edge of the aDMPFC, and 5 detectors were linearly placed over the aDMPFC from the source. The distance between each pair of the neighboring probes was 6 mm. The midpoint between the source and detector was similarly regarded as the “fNIRS channel,” and hemodynamic responses under the 5 channels (Ch 1–5) were detected by the 5 source-detector pairs using the same fNIRS instruments. In each channel, the changes in Hb concentration [ΔOxy-Hb, ΔDeoxy-Hb, and ΔTotal-Hb (ΔOxy Hb + ΔDeoxy Hb)] from the baseline level were estimated. Furthermore, a probe of the laser Doppler tissue blood flow meter (laser Doppler ALF 21, ADVANCE CO., LTD, Tokyo, Japan) (time constant, 0.1 s) was placed 20 mm below the line of the fNIRS probes. Data were sampled at 40 Hz.

After fNIRS recording, the 3-D locations of the probes were measured by a Digitizer (Real NeuroTechnology Co. Ltd., Japan) in reference to the nasion and both the external auditory meatus. The location of each fNIRS channel was determined by converting the actual coordinates to MNI coordinates (Ye et al., 2009).

### Data analyses in Experiment I

Hemodynamic responses were analyzed in 8 regions of interest (ROIs): the aDMPFC (a dorsomedial part of the area 10), SMA, left and right dorsal premotor area (L-PM, R-PM), left and right hand areas of the primary motor cortex (L-MI, R-MI), and left and right hand areas of the primary somatosensory cortex (L-SI, R-SI) (Figure 2B). Appropriate channels were selected for each ROI according to the MNI coordinates of the channels. Three hemodynamic response parameters (changes in Oxy-Hb, Deoxy-Hb, and Total-Hb) are assessed by fNIRS. These fNIRS data were summed and averaged in reference to the onset of each stimulation block. These averaged responses were corrected for baseline activity at the onset of the task phase. In the present study, we focused on changes in Oxy-Hb, which is sensitive to neurohemodynamic relationships in fNIRS studies (Hoshi et al., 2001; Strangman et al., 2002; Yamamoto and Kato, 2002). The hemodynamic response latency in each channel was defined as the interval from the onset of the task phase to the time at which the Oxy-Hb exceeded the mean ± 2.0 SD of the baseline level. The slope of initial rise of the hemodynamic responses was defined as the highest value among the instantaneous slopes within 15 s after the onset of the task phase. The instantaneous slopes of the hemodynamic responses were estimated by differentiation of the hemodynamic response curve. Response latencies and slopes of the hemodynamic responses were compared among the channels using the Wilcoxon signed rank test (*P* < 0.05) because the data were not normally distributed.

The above analyses indicated that the mean response latency was the shortest in the aDMPFC, and hemodynamic responses in the aDMPFC during the peg task gradually increased in later blocks (see Results). To quantify the increments in hemodynamic responses across the 8 blocks, the hemodynamic responses for each subject were analyzed using simple linear regression analysis. “Oxy-Hb gain” was defined as a slope of this regression line. We similarly analyzed peg score, which was defined as the number of the pegs put into the hole. “Performance gain” was defined as a slope of the regression line. The relationship between Oxy-Hb gain and performance gain were analyzed using simple linear regression analyses.

### Data analyses in Experiment II

For subjects who were fitted with the linear head cap, the data from the 5 channels were analyzed. These fNIRS data (changes in Oxy-Hb, Deoxy-Hb, and Total-Hb) were summed and averaged in reference to the onset of each stimulation block. These averaged responses were corrected for baseline activity at the onset of the task phase. Correlations between laser Doppler signals and fNIRS signals (Oxy-Hb) were analyzed using Pearson's correlation coefficient.

We attempted to dissociate between the brain and scalp hemodynamic responses using multi-distance probe arrangement (using the linear head cap) (Saager and Berger, 2008; Yamada et al., 2009). fNIRS signals from Ch1 with 6.0 mm source-detector distance are supposed to reflect hemodynamic activity confined within the scalp, whereas fNIRS signals from the extra-scalp layers, such as the brain, gradually increase in channels with larger source-detector distance (i.e., Ch 2–5) (Kohri et al., 2002). That is, fNIRS signals in channels with larger source-detector distances include both hemodynamic responses in the scalp and other layers, including the brain. As previously reported (Saager and Berger, 2008; Yamada et al., 2009), fNIRS signals from the extra-scalp layers in i-th channel at time t, Ci-[Oxy-Hb](t), were estimated as:

$$\begin{array}{l} {\text{C}}_{\text{i}}-[\text{Oxy}-\text{Hb}](\text{t})=[\text{Oxy}-\text{Hb}]{\left(\text{t}\right)}_{\text{Ch }-\text{ i}}\\ \;-{\text{K}}_{\text{i}}\cdot [\text{Oxy}-\text{Hb}]{\left(\text{t}\right)}_{\text{Ch }-\text{ 1}} \end{array}$$

where [Oxy-Hb](t)Ch-i is Oxy-Hb concentration derived from the signals in i-th channel at time t, and K_i_ (*i* = 2 to 5) is scaling constant that denotes the ratio of the scalp-derived hemodynamic responses (hemodynamic responses in Ch1) contributing to the hemodynamic responses in Ch-i. To estimate the constant “K_i_,” we performed an independent component analysis (ICA) using the algorithm proposed by Molgedey and Schuster (1994). The time delay was set at 100 samples at 40-Hz sampling rate. This ICA algorithm decomposes n-channel data into n independent components, each of which corresponds to a recovered putative source that contributes to fNIRS signals. This process is noted as the following equation:

$$\left(\begin{array}{c} {\text{X}}_{1}(\text{t})\\ {\text{X}}_{2}(\text{t})\\ \cdot \\ \cdot \\ {\text{X}}_{n}(\text{t}) \end{array}\right)=\left(\begin{array}{ccccc} a_{11} & a_{12} & \cdot & \cdot & a_{1n}\\ a_{21} & a_{22} & \cdot & \cdot & a_{2n}\\ \cdot & \cdot & \cdot & \cdot & \cdot \\ \cdot & \cdot & \cdot & \cdot & \cdot \\ a_{n1} & a_{n2} & \cdot & \cdot & a_{nm} \end{array}\right)\left(\begin{array}{c} {\text{S}}_{1}(\text{t})\\ {\text{S}}_{2}(\text{t})\\ \cdot \\ \cdot \\ {\text{S}}_{n}(\text{t}) \end{array}\right)$$

where X_i_(t) is NIRS signals (changes in absorbance (ΔAbs) of light in the 3 wavelengths [780, 805, 830 nm]) from Ch-i at time t, S_i_(t) is signal from i-th independent component at time t, and *a*_nj_ denotes the mixing coefficient in the mixing matrix.

Then, scalp-derived hemodynamic responses in each channel were simulated. We assumed that signals from the brain gray matter were dependent on source-detector distance, whereas signals from the scalp were not dependent on source-detector distance (Kohri et al., 2002). To estimate dependency on source-detector distance, mixing coefficients *a*_ij_ in i-th column of the mixing matrix were analyzed using simple regression analyses. When the absolute value of the slope of the resultant regression line was greater than 0.01, the mixing coefficients *a*_ij_ in i-th column were considered to be distance-dependent. In such cases, weight coefficient *w*_ij_ was introduced for each mixing coefficient so that the slope of the regression line for *w*_ij_a_ij_ became zero. When the absolute value of the slope of the resultant regression line was smaller than 0.01, the mixing coefficients *a*_ij_ in i-th column were considered to be distance-independent, and *w*_ij_ was set at 1. By introducing the weight coefficient *w*_ij_, the scalp-derived signal, X′_i_(t), in each channel was estimated as:

$$\left(\begin{array}{c} {\text{X}}^{\prime }{}_{1}(\text{t})\\ {\text{X}}^{\prime }{}_{2}(\text{t})\\ \cdot \\ \cdot \\ {\text{X}}^{\prime }{}_{n}(\text{t}) \end{array}\right)=\left(\begin{array}{ccccc} w_{11}a_{11} & w_{12}a_{12} & \cdot & \cdot & w_{1n}a_{1n}\\ w_{21}a_{21} & w_{22}a_{22} & \cdot & \cdot & w_{2n}a_{2n}\\ \cdot & \cdot & \cdot & \cdot & \cdot \\ \cdot & \cdot & \cdot & \cdot & \cdot \\ w_{n1}a_{n1} & w_{n2}a_{n2} & \cdot & \cdot & w_{nm}a_{nm} \end{array}\right)\left(\begin{array}{c} {\text{S}}_{1}(\text{t})\\ {\text{S}}_{2}(\text{t})\\ \cdot \\ \cdot \\ {\text{S}}_{n}(\text{t}) \end{array}\right)$$

Based on the above equation, simulated scalp-derived fNIRS signals in Ch-i, X′_i_(t), were reconstructed. Next, the scaling constant K_i_ (*i* = 2 to 5) was estimated as;

$${\text{D}}_{\text{i}}(\text{t})={\text{X}}^{\prime }{}_{\text{i}}(\text{t})-{\text{K}}_{\text{i}}\cdot {\text{X}}_{1}(\text{t})$$

where K_i_ is a value that minimizes the root mean square of D_i_(t). We used fNIRS signals from Ch1 as reference fNIRS signals from the superficial (scalp) layer, and the hemodynamic response corrected for its scalp-derived component was estimated in Ch 2–5. In Experiment II, the K_i_ value for each Ch 2–5 was estimated in each of the 3 tasks (resting, reaching, and peg tasks) for each subject. Because there were no significant differences in mean K_i_ values among the 3 tasks for Ch 2–5 (see Results), K_i_ values derived from the data in the resting task were used to correct hemodynamic responses in the reaching and peg tasks for their scalp-derived components.

### Experimental protocol of tDCS in Experiment III

The subjects were required to perform the same peg task with and without tDCS before the task. A specially developed battery-driven constant current stimulator (DC-stimulator Plus, Neuroconn, Ilmenau, Germany) was used. One anodal electrode (surface area 35 cm^2^) was positioned over the aDMPFC. The cathode was placed on the occipital area (2 cm above the inion). The 14 subjects were pseudo-randomly assigned to 2 groups: 7 subjects in the tDCS group and 7 subjects in the sham group. In the tDCS group, constant current (1.0 mA) was delivered between 2 sponge surface electrodes soaked with sodium chloride for 900 s. In the sham group, the same electrodes were placed in the same positions, and the same current was delivered only for initial 30 s. The current density was 0.0000285 A/cm^2^, and the amount of current (the current density × duration) was 0.02565 C/cm^2^, which was below the international criteria (0.03 Asec/cm^2^) (Nitsche et al., 2003b). Peg scores in the 2 groups were compared using repeated measures Two-Way analysis of variance (ANOVA). Statistical significance was set at *P* < 0.05.

## Results

### Experiment I

#### Behavioral data

The mean peg scores gradually increased across the 8 blocks. A simple regression analysis indicated that mean page score positively correlated with block number [*r*^2^ = 0.145, *F*_(1, 94)_ = 15.986, *P* < 0.01]. Statistical analyses using repeated measures One-Way ANOVA indicated there was a significant main effects of block [*F*_(7, 77)_ = 8.657, *P* < 0.001]. *Post-hoc* tests indicated that mean peg scores in blocks 6–8 were higher than those in blocks 1–2 (Tukey's test, *P* < 0.05).

#### Hemodynamic responses during the peg task

**Figure 3:**
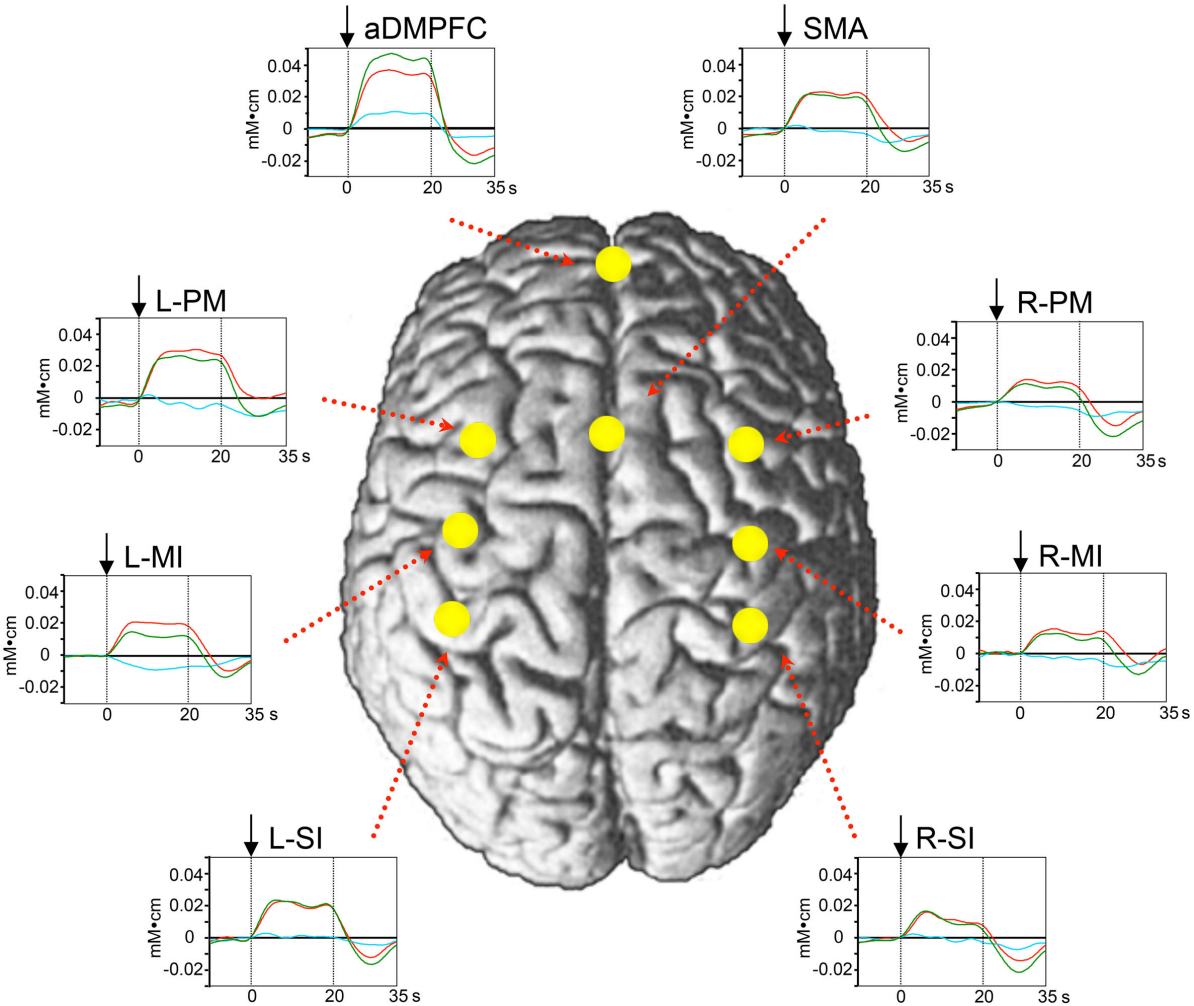
**Averaged hemodynamic responses in the peg task from Experiment I**. Red, green, and blue lines indicate changes in Oxy-Hb, Total-Hb, and Deoxy-Hb, respectively. Changes in Oxy-Hb rapidly increased during the task phase at the 8 ROIs. Arrows indicate the onset of the task phase. The other descriptions are the same as those for Figure 1.

**Figure 4:**
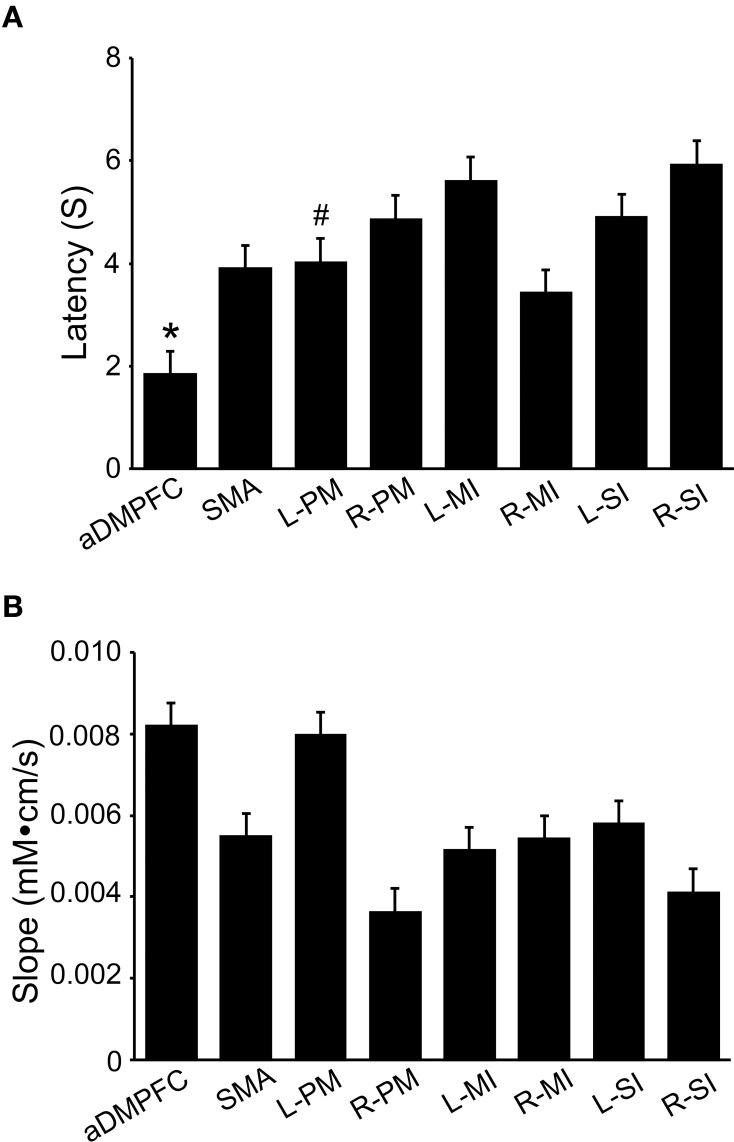
**Comparison of latencies (A) and slope (B) of hemodynamic responses (Oxy-Hb) among the 8 ROIs**. Responses latencies were significantly shorter in the aDMPFC than the other ROIs, except the L-PM (Wilcoxon signed rank test, *P* < 0.05), and tended to be shorter in the aDMPFC than in the L-PM (Wilcoxon signed rank test, *P* < 0.1). ^*^P < 0.05 compared to other areas, except the L-PM; #*P* < 0.1 trend difference from the aDMPFC **(A)**. The other descriptions are the same as those for Figure 1. However, there was no significant difference in the slope among the ROIs (Wilcoxon signed rank test, *P* > 0.05) **(B)**.

Figure 3 shows mean hemodynamic responses in the 8 ROIs. Oxy-Hb concentration increased during the peg task phase in the 8 ROIs. Figure 4 shows the mean response latencies and slope of the hemodynamic responses in the 8 ROIs. The response latencies were significantly shorter in the aDMPFC compared to the other ROIs, except the L-PM (Wilcoxon signed rank test, *P* < 0.05), and tended to be shorter than that in the L-PM (Wilcoxon signed rank test, *P* < 0.1) (A). However, there was no significant difference in the slope among the ROIs (Wilcoxon signed rank test, *P* > 0.05) (B).

**Figure 5:**
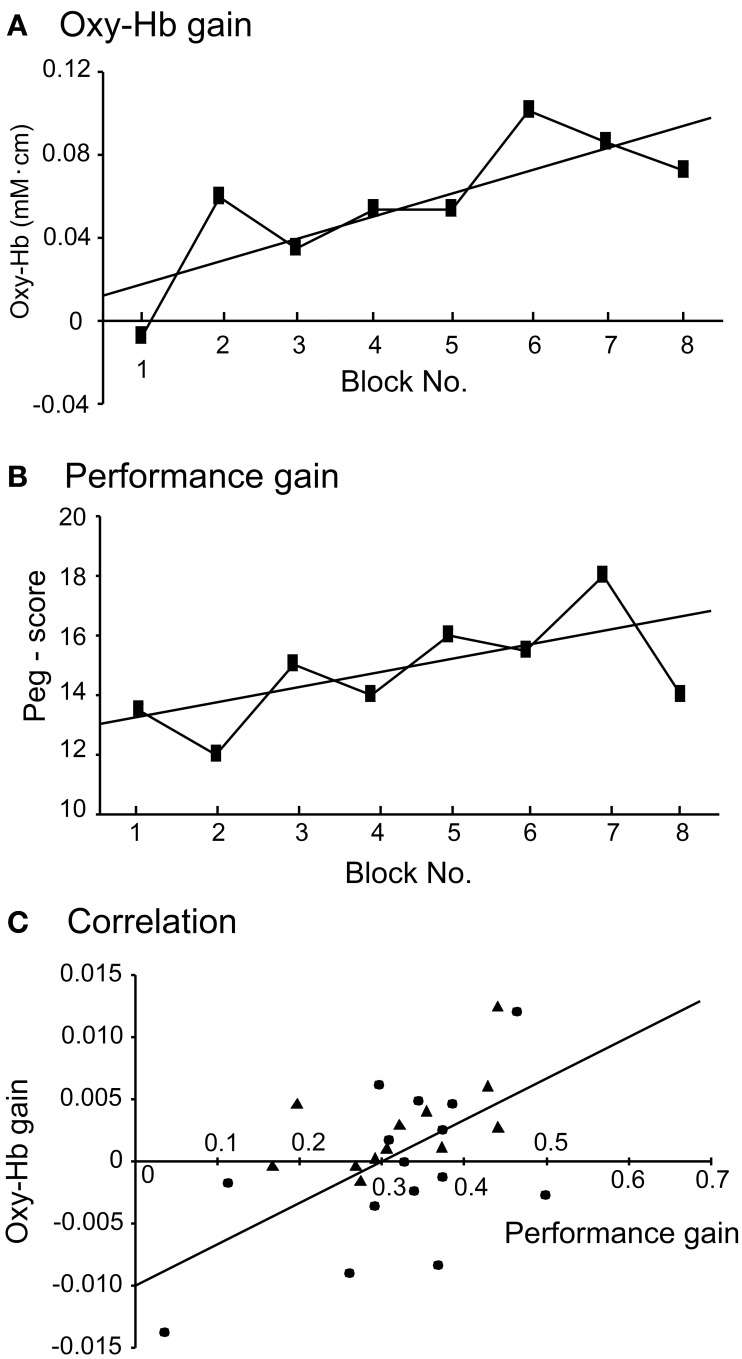
**Relationships between task performance and hemodynamic responses in the aDMPFC. (A)** Examples of changes in hemodynamic responses (Oxy-Hb) across the 8 blocks in 1 subject. The slope of the regression line was defined as “Oxy-Hb gain.” **(B)** Examples of changes in peg score across the 8 blocks in the same subject. The slope of the regression line was defined as “performance gain.” **(C)** Significant relationships between Oxy-Hb gain and performance gain. Filled triangles and circles indicate the subjects in Experiments I and II, respectively. The line indicates the regression line in the combined data from Experiments I and II.

The above data suggest that aDMPFC activity increased before activity in the other areas (but, see Discussion) and that the aDMPFC may facilitate motor learning through the sensorimotor areas. Next, we analyzed whether performance variance of individuals correlated with individual differences in hemodynamic responses in the aDMPFC. To analyze this relationship, we analyzed “Oxy-Hb gain” and “performance gain” in each subject. Examples of changes in Oxy-Hb concentration and performance across the 8 blocks in 1 subject are shown in Figures 5A,B; the slopes of the regression lines of these data indicate Oxy-Hb gain and performance gain, respectively. The relationship between Oxy-Hb gain and performance gain is shown in Figure 5C (filled triangles). A simple regression analysis revealed a significant positive correlation between Oxy-Hb gain and performance gain [*r*^2^ = 0.333, *F*_(1, 10)_ = 4.989, *P* < 0.05]. Furthermore, Oxy-Hb gain in the aDMPFC significantly and positively correlated with that in the L-PM, R-PM, L-MI, L-SI, and R-SI (Pearson's correlation coefficients, *P* < 0.05). These indicate that performance improvement might be attributed to hemodynamic changes in the aDMPFC, which might modulate other motor-related areas to facilitate motor learning. Therefore, we further investigated hemodynamic responses in the aDMPFC in Experiment II.

### Experiment II

#### Hemodynamic responses during the reaching and peg tasks

**Figure 6:**
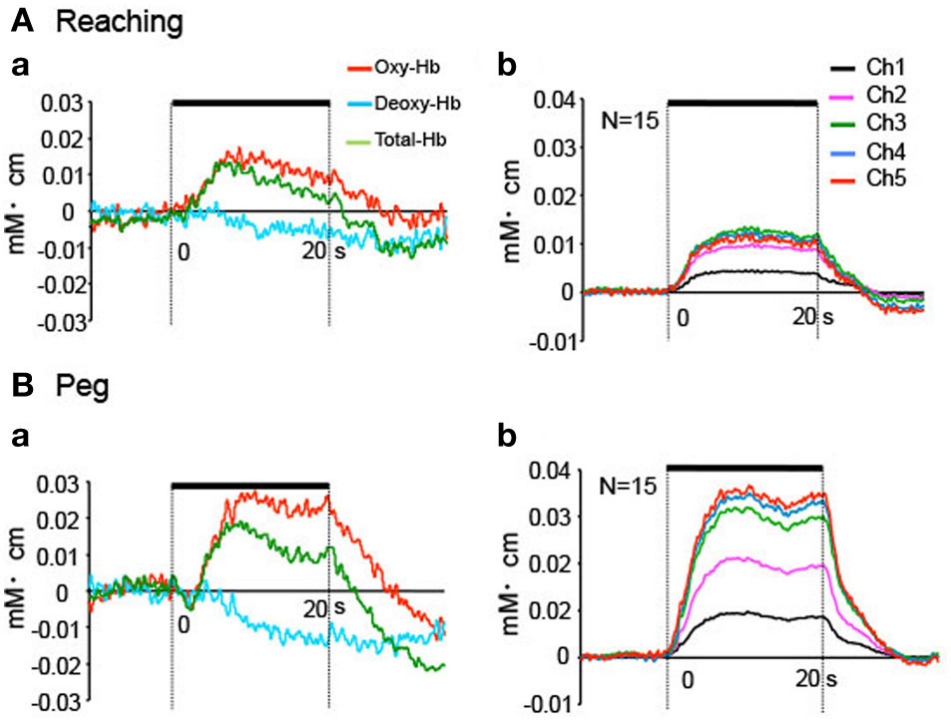
**Hemodynamic responses during the task phase in Experiment II. (A)** Hemodynamic changes in the reaching task in 1 subject **(a)** and the averaged data of 8 repetitions (all blocks) for all subjects **(b)**. Only Oxy-Hb data are indicated in **(b)**. **(B)** Hemodynamic changes in the peg task in 1 subject **(a)** and the averaged data of 8 repetitions (all blocks) for all subjects **(b)**. Only Oxy-Hb data are indicated in **(b)**. Thick lines indicate the task phase.

**Figure 7:**
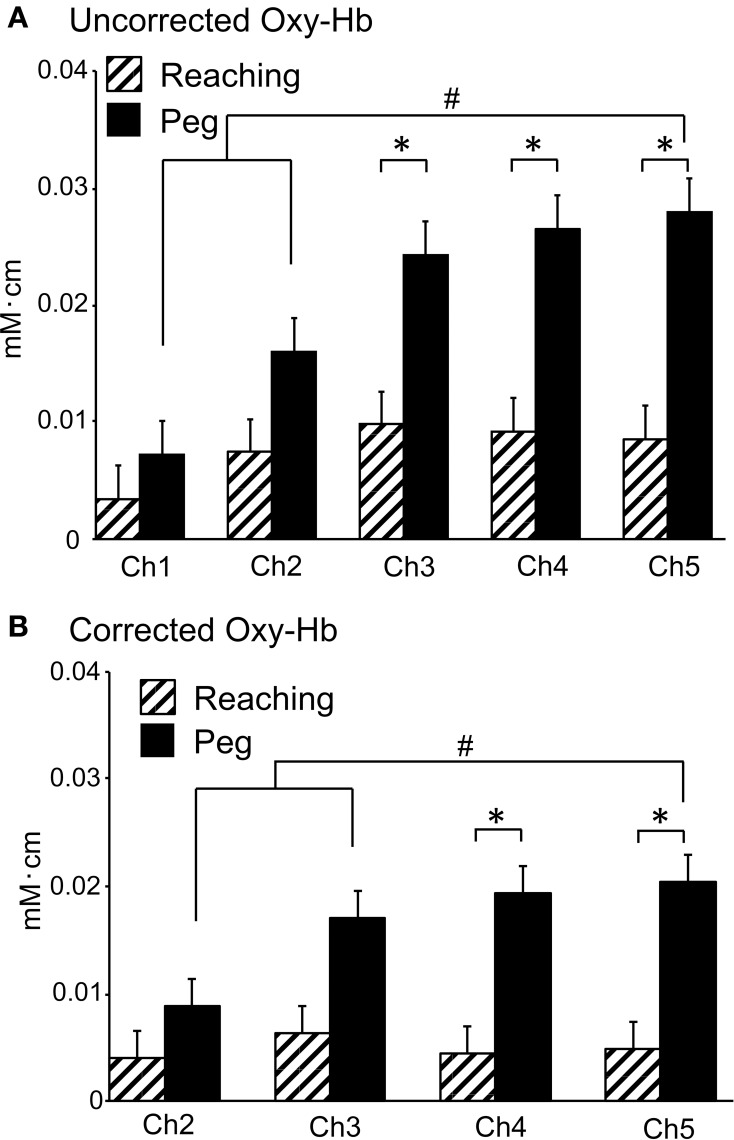
**Averaged hemodynamic responses (Oxy-Hb) in Experiment II. (A)** Averaged uncorrected hemodynamic responses. ^*^P < 0.05 compared to the reaching task; #*P* < 0.05 compared to Ch 5. **(B)** Averaged corrected hemodynamic responses. ^*^P < 0.05 compared to the reaching task; #*P* < 0.05 compared to Ch 5. Note that mean contribution ratio of the brain to hemodynamic signals at 3 cm of probe distance (Ch 5) was 66.6% according to the data in the peg task [i.e., corrected Oxy-Hb (B)/uncorrected Oxy-Hb (A) in Ch 5].

Figure 6 shows hemodynamic responses in the 5 fNIRS channels during the reaching and peg tasks. The data from Ch 5 in 1 subject indicated that changes in Oxy-Hb and Total-Hb concentration increased, whereas changes in Deoxy-Hb concentration gradually decreased during the reaching and peg tasks (Aa, Ba). Group-averaged data from Ch 1–5 indicated that changes in Oxy-Hb concentration increased during the reaching and peg tasks (Ab, Bb). In the reaching task, peak responses of Oxy-Hb concentration increased from Ch 1 to 2, but those in Ch 2–5 showed responses similar to those in Ch 2 (Ab). In the peg task, peak responses of Oxy-Hb concentration increased gradually from Ch 1 to 5 (Bb). Figure 7A shows mean hemodynamic responses (changes in Oxy-Hb) during the reaching and peg tasks. Repeated measures Two-Way ANOVA indicated that there were significant main effects of task [*F*_(1, 14)_ = 5.726, *P* < 0.05] and channel [*F*_(4, 56)_ = 9.508, *P* < 0.05] and significant interactions between task and channel [*F*_(4, 56)_ = 5.095, *P* < 0.01]. *Post-hoc* tests indicated that in the peg task, hemodynamic responses were significantly larger in Ch 5 compared to Ch 1–2 (Tukey's test, *P* < 0.05) and that hemodynamic responses in Ch 3–5 were significantly larger in the peg task than in the reaching task (Tukey's test, *P* < 0.05). These results indicate that hemodynamic responses in the aDMPFC were larger in the probe pairs with longer distances, as well as in the peg task. However, the fNIRS signals (Oxy-Hb) from all channels (Ch 1–5) significantly and positively correlated with the laser Doppler signals (*P* < 0.05) (Table 1). This indicates that the fNIRS signals, even for Ch 5, included hemodynamic activity in the scalp.

**Table 1:** **Correlation coefficients between NIRS channel pairs and between NIRS channel and laser Doppler signals (Doppler)**.

	**Ch1**	**Ch2**	**Ch3**	**Ch4**	**Ch5**
Ch2	0.998				
Ch3	0.996	0.996			
Ch4	0.996	0.997	0.998		
Ch5	0.994	0.996	0.995	0.997	
Doppler	0.922	0.932	0.928	0.933	0.937

*Note that fNIRS signals (Ch1–5) were highly correlated with laser Doppler signal*.

**Figure 8:**
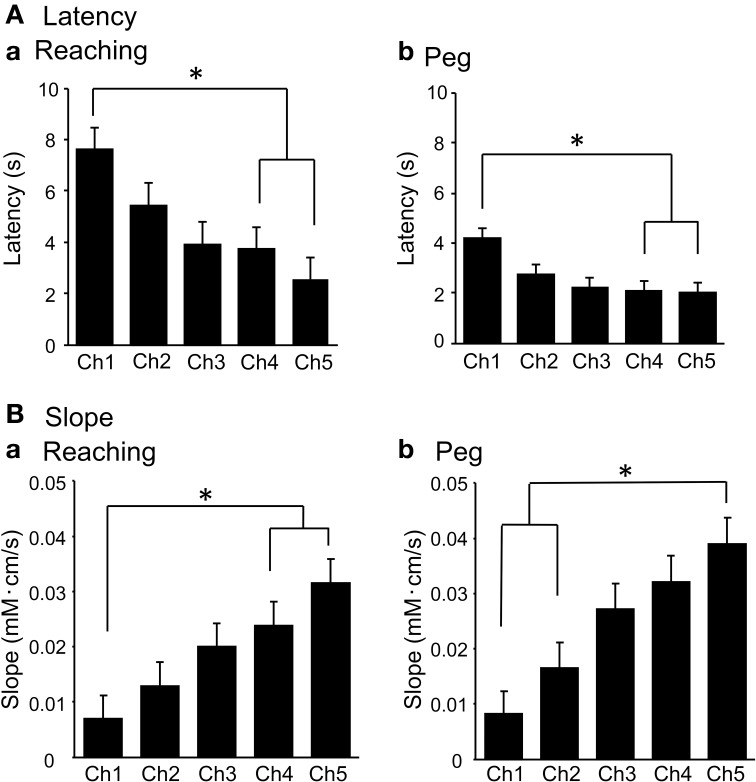
**Mean response latencies (A) and slopes (B) of the initial rise of the hemodynamic responses (Oxy-Hb) in the reaching (a) and peg tasks in Experiment II**. ^*^P < 0.05 compared to Ch 1.

Figure 8A shows mean latencies of the above hemodynamic responses. Repeated measures One-Way ANOVA indicated that during the reaching task, there were significant differences in mean latencies among the 5 channels [*F*_(4, 56)_ = 8.308, *P* < 0.01] (Aa). *Post-hoc* tests indicated that the mean latencies were significantly shorter in Ch 4–5 compared to Ch 1 (Tukey's test, *P* < 0.05). During the peg task, similar results were observed: repeated measures One-Way ANOVA indicated that there were significant differences in mean latencies among the 5 channels [*F*_(4, 56)_ = 11.490, *P* < 0.01] (Ab). *Post-hoc* tests indicated that the mean latencies were significantly shorter in Ch 4–5 than in Ch 1 (Tukey's test, *P* < 0.05). Figure 8B shows the mean slopes of the hemodynamic responses. Repeated measures One-Way ANOVA indicated significant differences in the mean slopes among the 5 channels during the reaching task [*F*_(4, 56)_ = 22.863, *P* < 0.01] (Ba). *Post-hoc* tests indicated that the mean slopes were significantly larger in Ch 4–5 compared to Ch 1 (Tukey's test, *P* < 0.01). In the peg task, similar results were observed and repeated measures One-Way ANOVA showed significant differences in the mean slopes among the 5 channels [*F*_(4, 56)_ = 21.195, *P* < 0.01] (Bb). *Post-hoc* tests indicated that the mean latencies were significantly larger in Ch 5 compared to Ch 1–2 (Tukey's test, *P* < 0.05). These results indicate that hemodynamic responses occurred more rapidly in Ch 4–5 than in Ch 1–2.

To correct the above hemodynamic responses for scalp-derived responses, K_i_ values were estimated for each channel and each task in individual subjects. For K_2_ values for Ch 2, repeated measures One-Way ANOVA showed no significant differences among the 3 tasks [*F*_(2, 28)_ = 1.212, *P* > 0.05]. For K_3_ values for Ch 3, repeated measures One-Way ANOVA indicated no significant differences among the 3 tasks [*F*_(2, 28)_ = 0.6485, *P* > 0.05]. For K_4_ values for Ch 4, repeated measures One-Way ANOVA showed no significant differences among the 3 tasks [*F*_(2, 28)_ = 0.7474, *P* > 0.05]. For K_5_ values for Ch 5, repeated measures One-Way ANOVA indicated no significant differences among the 3 tasks [*F*_(2, 28)_ = 1.445, *P* > 0.05]. Thus, there were no significant differences in Ch 2–5 K_i_ values among the 3 tasks. Therefore, the hemodynamic responses in Ch 2–5 were corrected for scalp-derived responses using K_i_ values estimated from the data in the resting task. Figure 7B shows the hemodynamic responses in Ch 2–5 corrected against the hemodynamic responses in Ch 1. Repeated measures Two-Way ANOVA showed significant main effects of task [*F*_(1, 14)_ = 5.644, *P* < 0.05] and channel [*F*_(3, 42)_ = 3.991, *P* < 0.05], indicating that the corrected hemodynamic responses were larger in the peg task than the reaching task. There was also a significant interaction between task and channel [*F*_(3, 42)_ = 5.274, *P* < 0.01]. *Post-hoc* tests indicated that the corrected hemodynamic responses in Ch 4–5 were larger in the peg task compared to the reaching task (Tukey's test, *P* < 0.05), and that the corrected hemodynamic responses in the peg task were larger in Ch 5 compared to Ch 2–3 (Tukey's test, *P* < 0.05). These results indicate that cerebral hemodynamic responses in the aDMPFC were larger in the peg task than in the reaching tasks.

#### Relationship between the hemodynamic responses and task performance

Figure 5C indicates the relationship between Oxy-Hb gain based on the uncorrected data in Ch 5 and performance gain in each subject (filled circles). A simple regression analysis revealed a significant positive correlation between the Oxy-Hb gain and performance gain [*r*^2^ = 0.312, *F*_(1, 13)_ = 5.886, *P* < 0.05]. When these data from Experiment II were combined with data from Experiment I, a simple regression analysis also revealed a significant positive correlation between the Oxy-Hb gain and performance gain [*r*^2^ = 0.285, *F*_(1, 25)_ = 9.964, *P* < 0.01]. Furthermore, there was a significant positive correlation between the Oxy-Hb gain based on the corrected data (Ch 5) in Experiment II and performance gain [*r*^2^ = 0.41, *F*_(1, 13)_ = 9.019, *P* < 0.05]. These results indicate that performance improvement might be attributed to hemodynamic changes in the aDMPFC.

### Experiment III

#### Effects of tDCS on motor learning in the peg task

**Figure 9:**
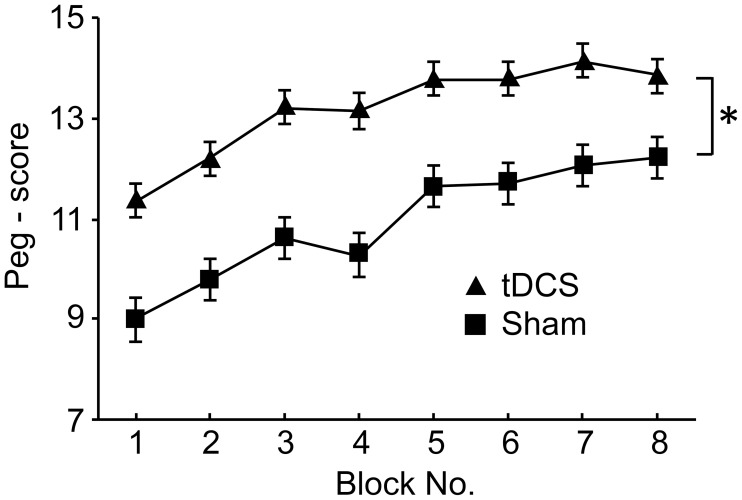
**Comparison of the peg scores between the tDCS and sham groups in Experiment III**. ^*^P < 0.05 between the peg scores of the 2 groups.

The subjects were required to perform the peg task with or without tDCS. Figure 9 shows changes in peg scores across the blocks. Peg scores gradually improved by repetition of the block in both the tDCS and sham groups. Repeated measures Two-Way ANOVA indicated significant main effects of group [*F*_(1, 6)_ = 6.248, *P* < 0.05] and block [*F*_(7, 42)_ = 15.358, *P* < 0.05], but no significant interaction between group and block [*F*_(7, 42)_ = 0.880, *P* > 0.05]. *Post-hoc* tests indicated that peg scores in blocks 7–8 were significantly larger than those in blocks 1–4 (Bonferroni test, *P* < 0.05), and that peg scores in block 1 were significantly smaller than those in blocks 2–8 (Bonferroni test, *P* < 0.05). These results indicated that repeated performance of the peg-task improved peg scores, and that tDCS significantly improved peg scores.

## Discussion

### Relationship between skin blood flow and cerebral hemodynamics

The present results indicate that hemodynamic responses in long-distance source-detector pairs (Ch 5) are significantly larger than those in short-distance source-detector pair (Ch 1), consistent with the findings of a previous study (Kohri et al., 2002; Ohmae et al., 2006; Saager and Berger, 2008). Monte Carlo simulations of a 5-layered tissue model indicated that signals from the superficial layer affect fNIRS signals and become the dominant signals in short source-detector pairs (0.2–0.4 cm) (Mansouri et al., 2010; Takahashi et al., 2011), and partial differential path-length factor for gray matter reflecting NIRS signal sensitivity to brain activation proportionally increases with increasing the source-detector spacing beyond 1.0 cm (Fukui et al., 2003). It has been reported that a source-detector pair with short distance (less than 1 cm) picks up the signal predominantly from the extracerebral layers such as the scalp, whereas a long distance source-detector pair picks up signals from the extracerebral as well as the intracerebral regions (Saager and Berger, 2008; Gagnon et al., 2011). The signals from the extracerebral layers can then be removed from the long distance source-detector probe (Saager and Berger, 2008; Gagnon et al., 2011).

Previous studies reported that fNIRS measurements using the multi-distance probe arrangement similar to the one used in the present study was effective in extracting hemodynamic responses from the gray matter (Saager and Berger, 2008; Yamada et al., 2009) and is normally referred to as the subtraction (linear regression) method. This subtraction method supposes that signals from the scalp and brain are dissimilar, and all signals similar to the scalp component are subtracted from signals from a long-distance probe pair (Saager and Berger, 2008). This method is useful because it estimates hemodynamic activity in the brain without requiring estimates of local scalp and skull thickness and scattering coefficients (Saager and Berger, 2008). However, a recent physiological study reported that signals from the scalp and brain include similar components (Moody et al., 2005). If the brain-derived component includes similar components from the scalp, the subtraction methods may overestimate the extra-brain component. We also applied the subtraction methods by Saager and Berger (2008) to the data in Ch1 and 5 in the peg task. The results indicated that the extra-brain component (i.e., Ch 1) accounted for about 60% of hemodynamic responses in the Ch 5 (data not shown), which is comparable to the results by Saager and Berger (2008). However, a previous study on the same prefrontal area at wavelength around 800 nm in a resting condition using a time-resolved spectroscopy and positron emission tomography (PET) reported that mean contribution ratio of the brain to hemodynamic signals at 3 cm of probe distance was about 65% (i.e., the extra-brain component accounted for 35% of the hemodynamic signals at 3 cm of probe distance) (Ohmae et al., 2006). These results suggest that the subtraction methods by Saager and Berger (2008) may overestimate the extra-brain component.

Even if the brain-derived component includes similar components from the scalp (Moody et al., 2005), their contribution to the signals becomes greater in longer distance source-detector pairs (Kohri et al., 2002). Therefore, we defined the components, the contribution ratio of which does not change across the signals from different distance source-detector pairs, as scalp-derived components. This method can theoretically dissociate brain-derived components even if signals of brain-derived components are similar to those of scalp-derived components. In the present study, mean contribution ratio of the brain to hemodynamic signals at 3 cm of probe distance (Ch 5) was 66.6% according to the data in the peg task in Figure 7 (i.e., corrected Oxy-Hb/uncorrected Oxy-Hb in Ch 5), which is comparable to those in Ohmae et al. (2006). However, further studies are required to determine whether the present results on brain contribution ratio and scaling constant K can be applied to other brain areas and other task conditions. Therefore, it should be noted that hemodynamic responses in the brain areas other than the aDMPFC in the Experiment I were uncorrected, and should be corrected using scaling constant K in each brain area in the future studies.

It is noted that the mean slopes of the initial rising phase of the hemodynamic responses were significantly larger in Ch 5 than in Ch 1 in the original (uncorrected) data, whereas the mean latencies were shorter in Ch 5 compared to Ch 1 in the original data. This indicates that the hemodynamic responses occurred faster in Ch 5 than in Ch 1, suggesting that hemodynamic responses in the aDMPFC occurred faster than those in the scalp. The aDMPFC, including the anterior cingulate cortex, has been implicated in control of various physiological responses including blood adrenocorticotropic hormone (ATCH) levels (Liberzon et al., 2007), the skin conductance response reflecting sympathetic activity (Critchley et al., 2000), and sympathetic activity (Yasui et al., 2010; Takamoto et al., 2013). Furthermore, an event-related fMRI study reported that activity of the aDMPFC preceded autonomic responses, suggesting that the aDMPFC is involved in generation of autonomic responses (Critchley et al., 2000). These findings are consistent with the idea that the initial rapid rise of the hemodynamic responses in Ch 5 might be attributed to cerebral hemodynamic responses in the aDMPFC, and the delayed hemodynamic responses in Ch 1 might be attributed to autonomic responses in the scalp induced by aDMPFC activity. However, it should be noted that latency and slope differences between Ch 5 and Ch 1 could be ascribed to differences in response magnitudes between Ch 5 and Ch 1; larger responses result in shorter latencies and larger slopes. Further studies would be necessary to investigate these possibilities.

### Role of the aDMPFC in motor learning

The data in Experiment I indicate that latencies of hemodynamic responses were shorter in the aDMPFC than other ROIs. However, latency differences among the ROIs could be ascribed to differences in response magnitudes among the ROIs, as discussed in the above section. Furthermore, Oxy-Hb gain in the aDMPFC significantly and positively correlated with that in the L-PM, R-PM, L-MI, L-SI, and R-SI. It has been proposed that the lateral frontal lobe is functionally organized along a rostral–caudal axis, and the subregions within the lateral frontal lobe including the aDMPFC, PM, MI, and SI are functionally interconnected (Petrides, 2005). These findings suggest that aDMPFC activity increases before activity in the other ROIs, and that the aDMPFC facilitates the other brain areas including the PM, MI, and SI.

The present study also indicates that hemodynamic responses in the aDMPFC significantly correlate with improved performance in the peg task. Furthermore, the corrected hemodynamic responses in Ch 5 in the aDMPFC were greater in the peg task, in which fine motor control (finger movements) was required, than in the reaching task, in which fine motor control was not required. However, gross movements of the upper limb were similar in both tasks. These data indicate that the aDMPFC is important in performance improvement. Non-invasive imaging studies suggest that the rostral part of the PFC, including the aDMPFC, was activated primarily when subjects learned new motor task(s) (Jenkins et al., 1994; Floyer-Lea and Matthews, 2004), and lesions in these prefrontal areas delayed motor learning (de Guise et al., 1999; Richer et al., 1999). Monkey neuroanatomical studies reported that the frontal pole, including the aDMPFC, projects to the dorsolateral and medial PFCs including the anterior cingulate cortex, and the striatum (Carmichael and Price, 1996; Petrides and Pandya, 2007), which are involved in motor learning (Halsband and Lange, 2006). aDMPFC activation during the peg task might facilitate these neural networks. Further, the medial PFC, including the aDMPFC, has intimate anatomical connections with the orbital cortex involved in motivational control of behaviors (Carmichael and Price, 1996) and has been implicated in volitional (intentional) control of various cognitive processes, including action planning (Zysset et al., 2002; Haggard, 2008). Hemodynamic responses in the aDMPFC during the peg task might reflect this volitional control because the subjects were required to perform the task as quickly as possible. Moreover, the anteromedial part of the PFC sends glutamatergic projections to the ventral tegmental area (Geisler et al., 2007), which in turn sends dopaminergic projections to the MI (Hosp et al., 2011). It is reported that dopamine in the MI facilitates motor skill learning (Molina-Luna et al., 2009; Hosp et al., 2011). Furthermore, the ventral tegmental area depolarizes resting membrane potentials of prefrontal neurons through dopamine (Lewis and O'Donnell, 2000). These findings suggest that the aDMPFC might improve task performance partly through the ventral tegmental area. Further studies are required to investigate whether the aDMPFC is involved in some or all of these possibilities.

The present study also indicates that tDCS of the aDMPFC increases performance. This evidence further supports the idea that the aDMPFC is involved in performance gain. It has been reported that anodal current during tDCS increased excitability in the neocortex (Liebetanz et al., 2002). This increased excitability is attributed to increased glutamatergic activity (Nitsche et al., 2003a; Clark et al., 2011) and decreased gamma aminobutyric acidergic activity (Stagg et al., 2011). Consistent with these findings, tDCS strengthens synaptic connections (Nitsche et al., 2003b, 2004) through long-term potentiation (Fritsch et al., 2010), which underlies the neurophysiological mechanisms of learning and memory (Bliss and Collingridge, 1993; Martin et al., 2000; Rioult-Pedotti et al., 2000). These findings suggest that tDCS enhanced cortical activity underneath the anode, which may have facilitated cognitive functions in the aDMPFC that supports motor learning, resulting in an increased performance during the peg task.

Previous studies reported that tDCS on the primary motor, premotor, and supplementary motor cortices facilitated motor learning (Boros et al., 2008; Nitsche et al., 2010; Reis and Fritsch, 2011; Vollmann et al., 2012), and has been applied for rehabilitation after stroke (Schlaug et al., 2008; Butler et al., 2013). In the present study, there was no interaction between group and block, and peg score was significantly increased from block 1 in the tDCS group. Similar enhancement of motor learning by tDCS has been reported; within-session performance improvement occurs, and continues over days (Nitsche et al., 2003a,b,c; Antal et al., 2004; Reis et al., 2009). This enhancement pattern suggests that preexisting synaptic machinery is strengthened by tDCS (Reis et al., 2009). The present results along with the previous results provide a new candidate brain area (aDMPFC) for tDCS in motor rehabilitation.

### Conflict of interest statement

Mr. Akihiro Ishikawa is an employee of the company, which made the NIRS apparatus used in the present study.
